# Detection of host pathways universally inhibited after *Plasmodium yoelii* infection for immune intervention

**DOI:** 10.1038/s41598-018-33599-1

**Published:** 2018-10-16

**Authors:** Lu Xia, Jian Wu, Sittiporn Pattaradilokrat, Keyla Tumas, Xiao He, Yu-chih Peng, Ruili Huang, Timothy G. Myers, Carole A. Long, Rongfu Wang, Xin-zhuan Su

**Affiliations:** 1 Malaria Functional Genomics Section, Laboratory of Malaria and Vector Research, National Institute of Allergy and Infectious Disease, National Institutes of Health, Bethesda, MD 20892-8132 USA; 2 State Key Laboratory of Medical Genetics, Xiangya School of Medicine, Central South University, Changsha, Hunan 410078 The People’s Republic of China; 3 Department of Biology, Faculty of Science, Chulalongkorn University, Bangkok, 10330 Thailand; 4 National Center for Advancing Translational Sciences, National Institutes of Health, Bethesda, MD 20892-8132 USA; 5 Genomic Technologies Section, Research Technologies Branch, National Institute of Allergy and Infectious Diseases, National Institutes of Health, Bethesda, MD 20892-8132 USA; 6 Center for Inflammation and Epigenetics, Houston Methodist Research Institute, Houston, TX 77030 USA

## Abstract

Malaria is a disease with diverse symptoms depending on host immune status and pathogenicity of *Plasmodium* parasites. The continuous parasite growth within a host suggests mechanisms of immune evasion by the parasite and/or immune inhibition in response to infection. To identify pathways commonly inhibited after malaria infection, we infected C57BL/6 mice with four *Plasmodium yoelii* strains causing different disease phenotypes and 24 progeny of a genetic cross. mRNAs from mouse spleens day 1 and/or day 4 post infection (*p.i*.) were hybridized to a mouse microarray to identify activated or inhibited pathways, upstream regulators, and host genes playing an important role in malaria infection. Strong interferon responses were observed after infection with the N67 strain, whereas initial inhibition and later activation of hematopoietic pathways were found after infection with 17XNL parasite, showing unique responses to individual parasite strains. Inhibitions of pathways such as Th1 activation, dendritic cell (DC) maturation, and NFAT immune regulation were observed in mice infected with all the parasite strains day 4 *p.i*., suggesting universally inhibited immune pathways. As a proof of principle, treatment of N67-infected mice with antibodies against T cell receptors OX40 or CD28 to activate the inhibited pathways enhanced host survival. Controlled activation of these pathways may provide important strategies for better disease management and for developing an effective vaccine.

## Introduction

Infection of malaria parasites triggers dynamic and complex immune responses, and a coordinated response is required for successful resolution of a malaria infection^1,2^. The innate immune response is the first line of the host defense against microbial infections, which also initiates and modulates subsequent adaptive immunity. Recognition of pathogen-associated molecular patterns (PAMPs) by pattern recognition receptors (PRRs) activates a series of signaling cascades, leading to production of proinflammatory cytokines such as IL-1β, TNF-α, IFN-α/β, IFN-γ, and chemokines that mediate migration of immune cells to the affected tissues^3,4^. However, excessive or chronic cytokine responses can cause damages to tissues or the immune system^4^. For example, chronic high levels of type I IFN (IFN-I) can lead to persistent lymphocytic choriomeningitis virus (LCMV) infection, while blockage of IFN-I signaling activates CD4+ T cells for viral clearance^5,6^. Highly elevated TNF-α was reported to contribute to cerebral malaria, although the exact mechanism remains unclear^7^. Activation of regulatory T cells (Treg) and production of IL-10 and TGF-β have been shown to play an important role in controlling inflammatory responses and to prevent immune-associated damages^2^. Additionally, many negative regulators such as SOCSs, RNFs and TRIMs are activated to control or inhibit signaling pathways to limit the magnitude and duration of the inflammatory cytokine response^4,8,9^. Recently, a specific γδ (Vδ 6.3) T cell population producing M-CSF, CCL5, and CCL3 was found to be critical for preventing parasite recurrence^10^. An optimal immune response would be one that can effectively control infection, but not so strong as to cause severe damage to the host^2,11^.

The types and levels of immune response are also dependent on host genetic background and parasite species or strains^12,13^, which may lead to contradictory results or conclusions regarding the mechanism of host response to malaria infections. For example, *Plasmodium y. nigeriensis* N67 (N67) triggers an early IFN-I response leading to suppression of parasitemia day 7 post infection (*p.i*), whereas its isogenic parasite *P. y. nigeriensis* N67C stimulates a strong inflammatory response resulting in host death by day 7 *p.i*.^12,14,15^. Infection of mice with *P. y. yoelii* 17XL (or YM) results in host death within 7 days due to high parasitemia, whereas mice infected with its isogenic strain *P. y. yoelii* 17XNL recover from infection^13,16–18^. Additionally, variation in host genetic background can also influence parasite growth pattern and host mortality^19,20^. Children aged 1–5 often present with severe symptoms such as severe anemia, coma, hyperlactatemia, and pulmonary oedema, whereas adults generally have mild infections, suggesting a role of prior exposure in disease severity^21^. Despite the differences in disease phenotypes and host immune responses, malaria infections also share common features such as fever and anemia as well as the underlying mechanism of host immune responses^1,2^. A common theme of elevated pro-inflammatory cytokine/chemokine responses and differential expression of genes in erythropoiesis, glycolysis, B cell activation, and immunoglobulin production in different malaria species or strains were reported in various microarray analyses^22–25^. However, our understanding of host immune responses to malaria infections is incomplete, particularly the pathways inhibited after malaria infection, which has impeded the development of an effective vaccine and disease treatment^26^.

In malaria endemic regions, many people are continuously infected by malaria parasites; however, the majority of infected adults do not experience any disease symptoms^27^. Malaria symptoms, particularly those of severe diseases, are likely due to over reaction of host immunity^21^. Production of pro-inflammatory cytokines such as IFN-γ and TNF-α have been shown to be critical for protection; however, over activation of T cells and cytokine production can also lead to severe disease^28,29^. Therefore, infected adult carriers in endemic areas are likely to develop certain mechanisms to control parasitemia, and at the same time, suppress some inflammatory responses that cause fever and other malaria symptoms. Identification of the inhibited immune pathways and proper activation of specific inhibited immune pathways may help the host clear parasites and facilitate development of an effective vaccine. Here we performed a comparative transcriptomic study of host responses to infections with four *P. yoelii* strains/subspecies (for simplicity, strain will be used) exhibiting different virulent phenotypes and 24 progeny of a genetic cross, with a major goal of discovering immune pathways inhibited after malaria infection. Unique and shared host response pathways as well as temporal changes in gene expression in mice day 1 (24 h) and/or day 4 (96 h) *p.i*. with the parasites were characterized. Dramatic differences in host response were observed day 1 after infection with different parasite strains. On the other hand, common pathways in response to all the parasites were observed day 4 *p.i*., including activation of mitotic roles of Polo-like kinase pathway and inhibition of Th1 and dendritic cell (DC) maturation pathways. Based on results from these analyses and as a proof of principle, we designed experiments to modulate selected inhibited immune pathways and showed improved host survival, which may provide an important avenue for developing strategies for better disease control and vaccine development.

## Results

### Strong IFN response in N67-infected mice and enrichment of hematopoiesis pathways in 17XNL-infected mice day 1 *p.i*

**Figure 1:**
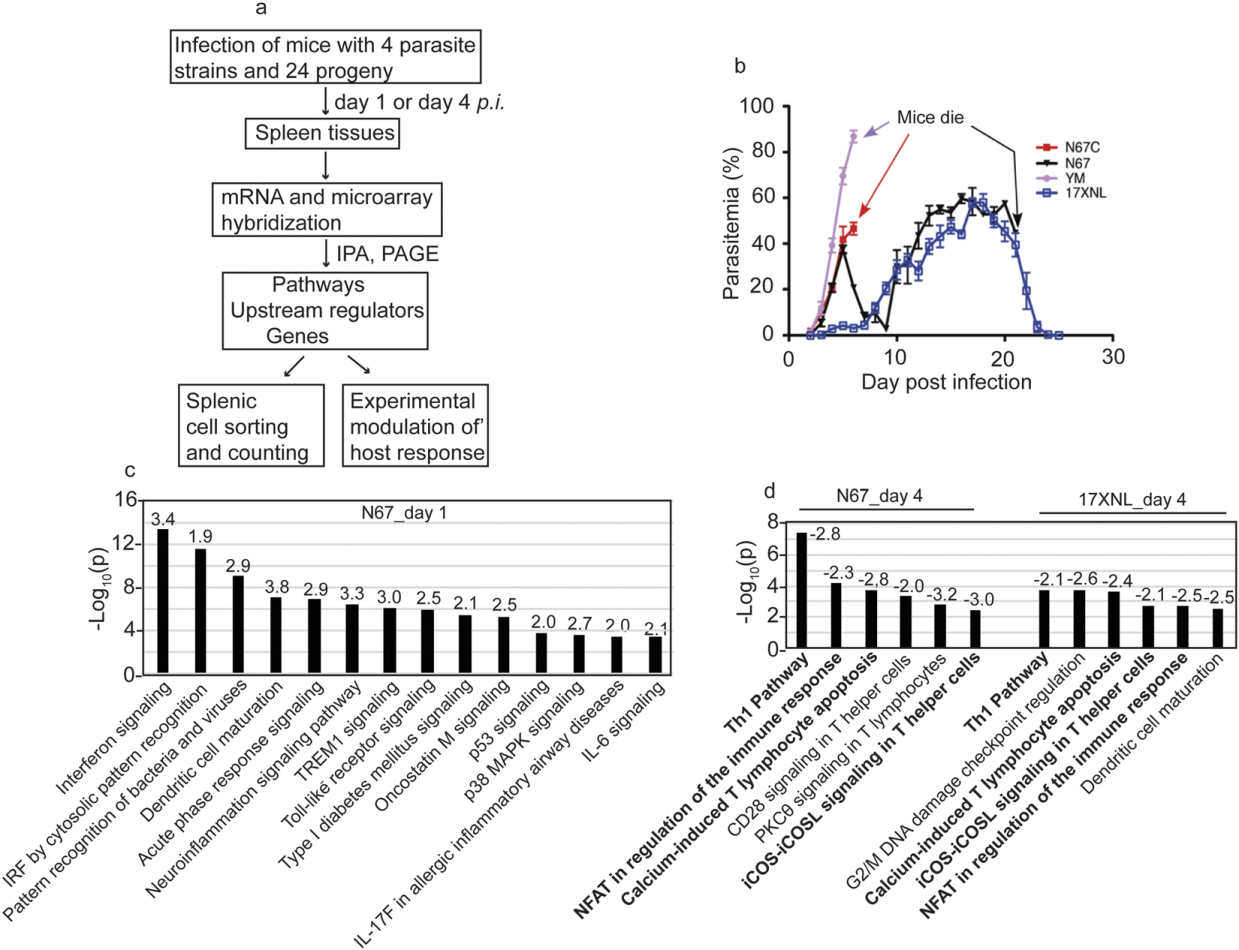
Significantly enriched activated or inhibited pathways after infection with N67 or 17XNL parasites. (**a**) Flowchart of experimental procedures conducted for this work. The methods and experimental procedures are as described in Materials and Methods. (**b**) Parasitemia curves from mice infected with four *Plasmodium yoelii* strains/subspecies. N67C, *P. y. nigeriensis* N67C; N67, *P. y. nigeriensis* N67; YM, *P. y. yoelii* YM; 17XNL, *P. y. yoelii* 17XNL. Arrows indicate time points of host death. C57BL/6 mice were injected *i.v*. with 10 × 10^6^ infected red blood cells (iRBCs) and daily parasitemia (percentage of iRBCs) were obtained after counting Giemsa stained thin blood smears. (**c**) Significantly enriched and activated pathways day 1 post infection with N67. The numbers on top of the bars are z-scores. A positive z-score suggests activation of a pathway. (**d**) Significantly and negatively enriched pathways day 4 post infection with N67 or 17XNL. The numbers on top of the bars are z-scores. A negative z-score indicates inhibition of a pathway. The pathway analyses were performed using Ingenuity Pathway Analysis (IPA).

To identify host pathways inhibited after malaria infection, we first hybridized mRNA samples from spleen tissues of mice (3–6 each group) infected with four *P. yoelii* strains and 24 progeny from a genetic cross following the procedures summarized in Fig. 1a. The four parasite strains grew differently in C57BL/6 mice and had different virulence phenotypes (Fig. 1b). For example, the parasitemia of N67 increased to approximately 40–50% on day 5, declined to below 5% on day 9, and then increased to 60–80% before killing the host on day 15–20 (Fig. 1b). The 17XNL grew slowly before day 10, reached to ~60% parasitemia on day 18, was cleared by the host on day 25 (Fig. 1b). We first compared gene expression levels between infected and uninfected mice using ANOVA and used a 2.0-fold cutoff value to generate gene lists. We then used Ingenuity Pathway Analysis (IPA, QIAGEN Inc., https://www.qiagenbioinformatics.com/products/ingenuity- pathway-analysis) to perform pathway analysis and to identify upstream regulators. From the differentially expressed gene lists, we identified pathways that were significantly (as indicated by p-value < 0.001) enriched in infected mice. We applied z-score value ≥ 2.0 for activated pathways and z-score ≤ −2.0 for inhibited pathways as recommended by the IPA package. For mice infected with N67 day 1 *p.i*., the top significantly enriched and activated pathways were those related to interferon signaling (Fig. 1c and Supplementary Fig. S1a), role of pattern recognition of bacteria and viruses, DC maturation, acute phase response signaling, neuroinflammation signaling pathway, TREM1 signaling, and Toll-like receptor signaling (Fig. 1c, Supplementary Tables S1, S2). Other pathways with significant gene enrichment, but without z-score ≥ 2.0, included role of hypercytokinemia and hyperchemokinemia in the pathogenesis of influenza (p = 3.2 × 10^−13^), activation of IRF cytosolic pattern recognition receptors (p = 2.5 × 10^−12^), and communication between innate and adaptive immune cells (p = 2.5 × 10^−12^) (Supplementary Tables S1, S2). LXR/RXR activation (p = 2.5 × 10^−4^) was the only significantly down-regulated pathway with a z-score = −2.83. These data suggest activation of various innate responses, particularly pathways of IFN signaling in early infection.

For mice infected with 17XNL day 1 *p.i*., no significantly activated or inhibited immune pathways were detected at 2-fold cutoff. However, activation of some molecules in the interferon pathways could be detected if we reduced the cutoff value to 1.2-fold (Supplementary Fig. S1b). Several heme-related biosynthesis pathways such as heme biosynthesis II (p = 1.2 × 10^−13^), tetrapyrrole biosynthesis II (p = 6.3 × 10^−7^), and heme biosynthesis from uroporphyrinogen III (p = 1.2 × 10^−7^) were significantly enriched and inhibited with z-scores ≤ −2.0 (Supplementary Tables S1, S2). G2/M DNA damage checkpoint regulation was the only pathway with a positive z-score (2.3). No immune related pathway significantly enriched (p < 0.001) and/or activated (z-score ≥ 2.0) was detected day 1 *p.i*. with 17XNL, which is probably due to slow parasite growth and low parasitemia.

### Inhibition of T cell pathways day 4 *p.i*. with both N67 and 17XNL

On day 4 *p.i*., the levels of IFN pathways were reduced in N67-infected mice; in particular, the expression levels of IFN-I and receptor for IFN-γ were further reduced, suggesting potential blockage of IFN signaling (Supplementary Fig. S2a). The pathway of mitotic roles of Polo-like kinase was the only one with a z-score ≥ 2.0, although pathways of altered T and B cell signaling in rheumatoid arthritis (p = 6.3 × 10^−11^), B cell development (p = 4.0 × 10^−10^), antigen presentation (p = 1.0 × 10^−9^), graft-versus-host disease signaling (p = 1.3 × 10^−9^), and Th1/Th2 activation (p = 6.3 × 10^−9^) were significantly enriched, but without a z-score (Supplementary Tables S1, S2). Many of the genes in these pathways were up-regulated day 1 *p.i*., but down-regulated day 4 *p.i*. (Supplementary Table S3), suggesting pathway inhibition day 4 *p.i*. There were also several inhibited pathways with z-score ≤ −2.0 such as Th1 pathway (p = 3.5 × 10^−8^), calcium-induced T lymphocyte apoptosis (p = 2.0 × 10^−4^), role of NFAT in regulation of the immune response (p = 5.8 × 10^−4^), and CD28 signaling in T helper cells (p = 4.1 × 10^−4^).

Similar to N67 infection, only the pathway of mitotic roles of Polo-Like kinase was significantly enriched and activated (p = 2.8 × 10^−6^ and z-score = 2.65) day 4 *p.i*. with 17XNL. Other enriched pathways including cell cycle control of chromosome replication (p = 2.0 × 10^−10^), B cell development (p = 8.5 × 10^−9^), antigen presentation pathway (p = 7.6 × 10^−8^), altered T and B cell signaling in rheumatoid arthritis (p = 7.8 × 10^−8^), and graft-versus-host disease signaling (p = 2.1 × 10^−4^) were also highly enriched, with most of the gene expression down-regulated day 4 *p.i*. (Supplementary Tables S1–S3). Interestingly, the pathway of G2/M DNA damage checkpoint regulation was enriched (p = 4.0 × 10^−6^) with a positive z-score (1.3) day 1 *p.i*., but was suppressed (p = 2.5 × 10^−4^; z-score = −2.45) at day 4 *p.i* with 17XNL (Supplementary Table S2). Significantly inhibited immune pathways with z-score ≤ −2.0 on day 4 *p.i*. again included Th1 pathway (p = 1.7 × 10^−4^) and calcium-induced T lymphocyte apoptosis (p = 1.8 × 10^−4^). iCOS-iCOSL signaling in T cell (P = 0.002), role of NFAT in regulation of immune response (p = 0.002), and DC maturation (P = 0.003) also had negative z-score ≤ −2.0, but the p-values for these pathways did not reach p < 0.001 (Fig. 1d and Supplementary Table S2). The expression of many molecules in the interferon pathways were also reduced day 4 *p.i*. with 17XNL (Supplementary Fig. S2b).

### Important genes differentially regulated between day 1 and day 4 *p.i*. with N67 and 17XNL parasites

**Figure 2:**
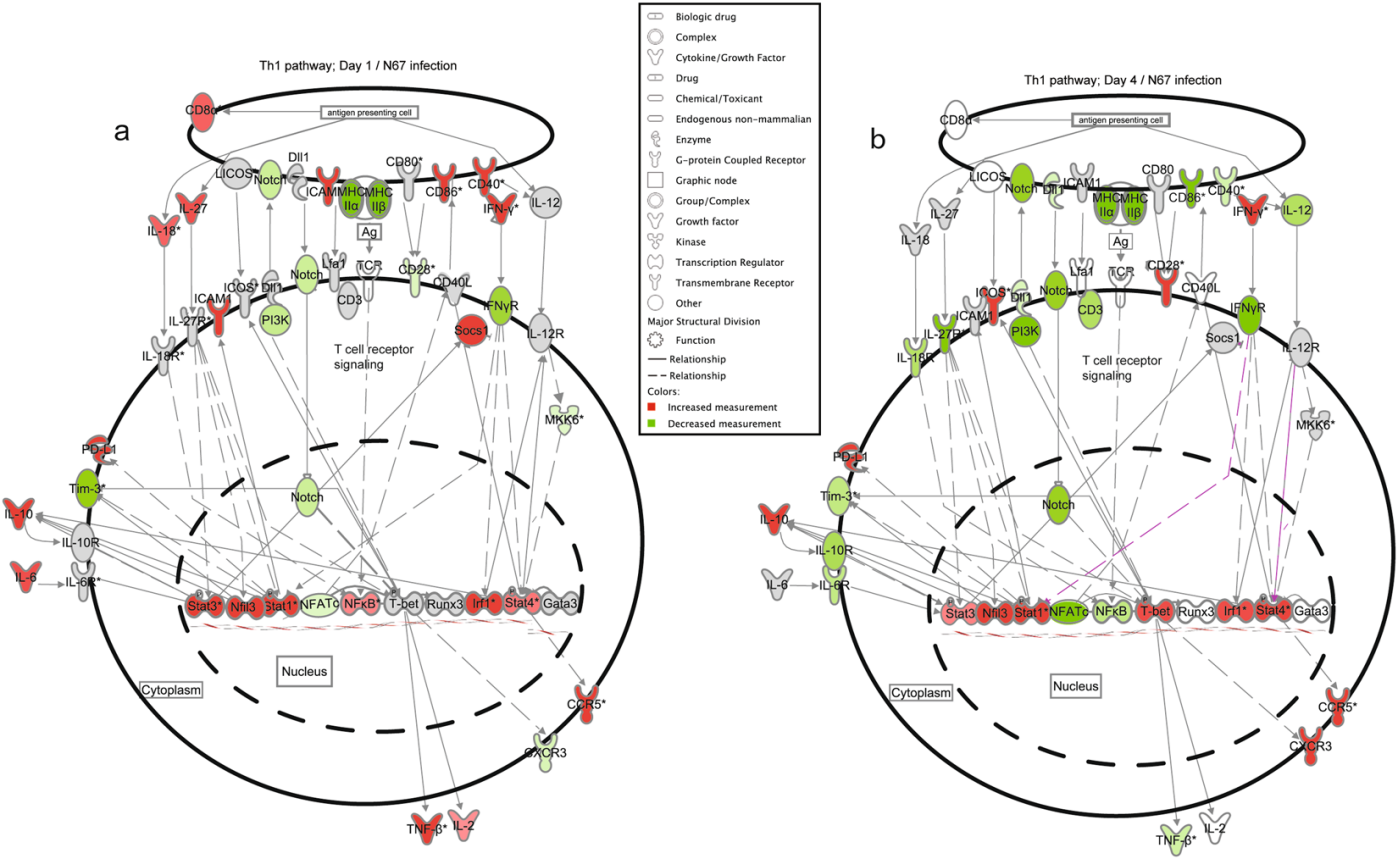
Inhibition of gene expression in the Th1 pathway in mice infected with N67. Genes differentially expressed in N67-infected and uninfected mice with a cutoff value of 1.2-fold or larger changes were selected for analysis of gene interaction networks that were generated through the use of IPA (QIAGEN Inc., https://www.qiagenbio informatics.com/products/ingenuity-pathway-analysis). Expression and interaction network of key genes in Th1 pathways day 1 (**a**) and day 4 (**b**) post infection with N67. Red, up-regulated genes; green, down-regulated genes. Darker colors suggest higher up- or down-regulated, respectively. Symbols for molecular functions are as presented in the side box. Note: many molecules in red (up-regulated expression) day 1 post infection became green (inhibited) or gray (similar to uninfected control) day 4 post infection.

Closer examination of individual gene transcripts in the Th1 pathway in mice day 1 *p.i*. with N67 showed highly up-regulated molecules such as *cd8α*, *cd40*, *cd86*, *icam1*, *cd274(pd-l1), irf1*, *stat1*, *stat3, nfκb*, *il-1β, il-6, and il-27* (Fig. 2a); however, most of these genes were de-activated or suppressed day 4 *p.i*. (Fig. 2a,b). In addition, many MHCII (major histocompatibility complex II) genes such as those encoding H2-Eb2, H2-DMa, H2-DMb1, H2-Oa, H2-Ob, H2-Aa, H2-Ab1 and H2-Eb1 were suppressed day 1 and further inhibited by day 4 *p.i*. (Supplementary Table S3). Interestingly, the transcript of the gene encoding T-bet, the master regulator of Th1 cell differentiation that controls the expression of IFN-γ^30^, was increased in infected mice from day 1 to day 4 *p.i* (Fig. 2a,b). Additionally, IFN-γ transcript was also increased in day 4 infected mice (Supplementary Table S3). The results suggest normal progression of T cell activation with ongoing inhibition of some specific T cell pathways, which may occur in different cell populations. The results also showed a clear shift in host immune responses, from innate activation of pattern recognition and interferon signaling day 1 *p.i*. to activation of adaptive immune responses as well as inhibition of specific T- and B-cell development pathways at day 4 *p.i*. The activation of early innate and adaptive immune response pathways, particularly IFN signaling, probably contributes to the decline of parasitemia from day 5 *p.i*. The suppression of T- and B-cell related immune pathways such as Th1 pathway, DC maturation (Supplementary Fig. S3a,b), and B cell development (Supplementary Table S3) after day 4 may contribute to the rebound of parasitemia after day 7^13^.

For mice day 1 *p.i*. with 17XNL, investigation of individual gene expression showed low-level activation of Th1 pathway (Supplementary Fig. S4a), and the majority of the genes did not express 2-fold higher than those of uninfected control. On day 4, the majority of the genes in the Th1 pathway were de-activated or suppressed, similar to those observed in N67-infected mice (Supplementary Fig S4b). In contrast, the expression of many genes in the mitotic roles of Polo-like kinase pathway were decreased day 1 *p.i*., but increased in mice day 4 *p.i*. (Supplementary Fig. S5a,b), suggesting activation of cell division. Similarly, many genes in the heme biosynthesis pathways and blood cell markers were slightly up-regulated day 4 *p.i*. (Supplementary Table S4), suggesting activation of hematopoiesis in the spleen from day 4 *p.i*.

### Activated and inhibited upstream regulators after infections with N67 and 17XNL

**Figure 3:**
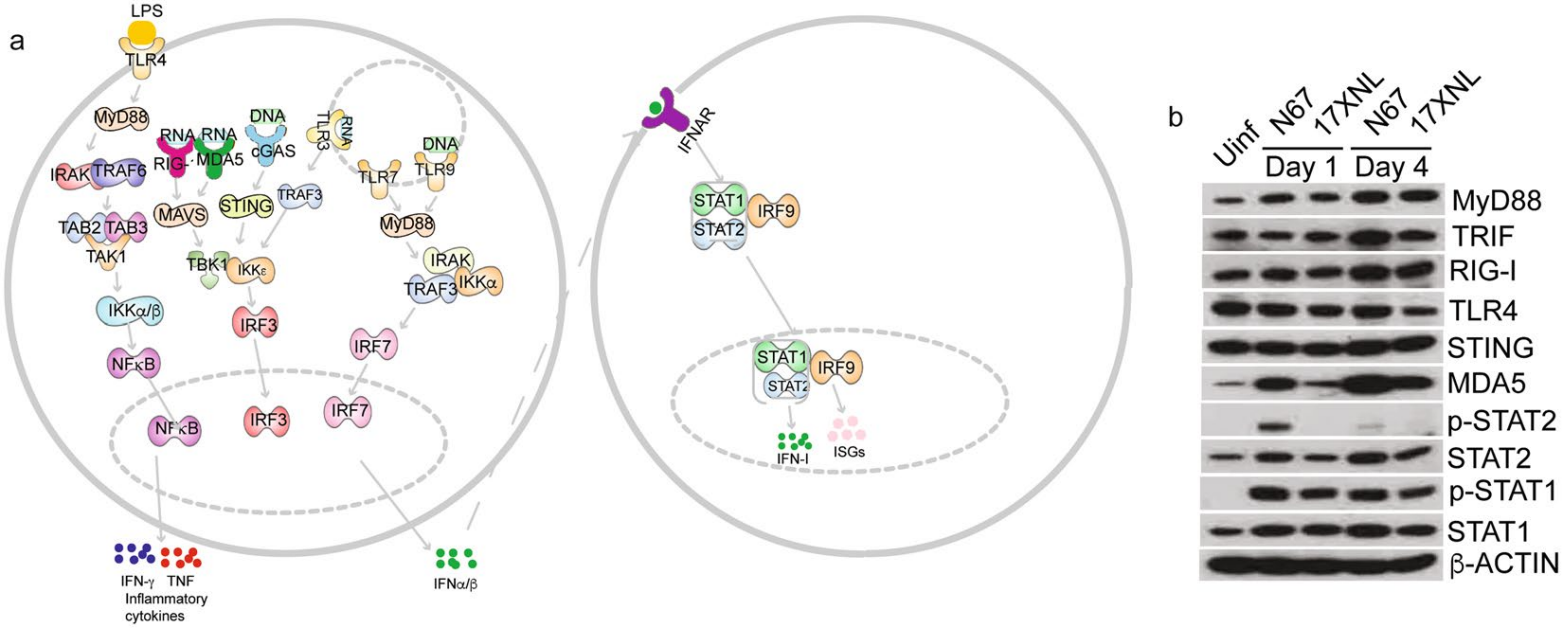
Activated top upstream regulators in mice infected with N67 and 17XNL *P. yoelii* parasites. Genes differentially expressed in N67- or 17XNL-infected and uninfected mice with a cutoff value of 2.0-fold or larger changes were selected for analysis using Ingenuity Pathway Analysis (IPA). (**a**) Signaling pathways derived from analysis of top upstream regulators after infection with N67 and/or 17XNL. TLR and cytosolic sensor mediated signaling pathways leading to production of IFN-I and pro-inflammatory cytokines (TNF and IFN-γ) are detected (or predicted such as LPS). Molecules shown are selected upstream regulators significantly activated (p < 0.001 and z-score ≥ 3.0) in mice day 1 post infection with N67 parasite as predicted by IPA analysis (also see Supplementary Table S5). (**b**) Western blot of protein expression and/or phosphorylation day 1 and day 4 after infection with N67 or 17XNL. Proteins from spleen tissues of infected mice were separated on SDS-PAGE gels, transferred to PVDF membrane, and probed with protein or phosphorylation specific antibodies after blotting to NC membrane. The full gel images for these Western blots are presented in Supplementary Fig. 6.

In addition to pathway analysis, we also investigated upstream regulators of signaling pathways to identify the cascade of transcriptional regulators that can explain the observed gene expression changes. The analysis of upstream regulators examines how many known targets of each transcription regulator are present in the dataset, and compares their direction of change to what is expected from the literature in order to predict probably relevant transcriptional regulators (http://pages.ingenuity.com/rs/ingenuity/images/0812%20upstream_regulator_analysis_whitepaper.pdf)^31^. We applied z-score value ≥ 2.0 for activated pathways and z-score ≤ −2.0 for inhibited pathways as recommended by the IPA package. Consistent with results from pathway analysis, recognition of RNA by TLR3/7, DNA by TLR9, and cytosolic pathways by cGAS and MDA5(IFIH1)/RIG-I (DDX58) were the top innate molecules significantly activated (activation z-score > 3.0 and p < 0.0001) in day-1 N67 infected mice, leading to activation of IRF3/7, STAT1/2, and IFNAR and production of IFN-α/β (Supplementary Table S5). Based on the highly activated upstream regulators in N67-infected mice day-1 *p.i*., some putative IFN-I signaling pathways can be constructed (Fig. 3a), which is consistent with the results from pathway analysis showing activated interferon pathways (Fig. 1a,c). Another activated pathway was TLR4 recognition of LPS and signaling through MyD88 and NFκB, leading to production of TNF, IFN-γ, and IL-1β (Fig. 3a and Supplementary Table S5). On day 4 *p.i*. with N67, although upstream regulators of LPS, TLRs, IRF7, NFκB, IL-1β, TNF-α/β, and IFN-γ were still present, the level of IFN-I signaling (z-scores) decreased, suggesting de-activation of IFN-I signaling and maintenance of inflammatory responses. Of interest, several negative regulators of IFN-I responses such as *mir21*, SOCS1, SOCS3, TRIM24^32–34^ as wells as IL10AR were inhibited day 1 *p.i*. with N67 (Supplementary Table S5). SOCS1, SOCS3, and TRIM24 remained suppressed day 4 *p.i*. with N67.

The upstream regulators after infection with 17XNL were more complex, involving molecules in diverse pathways. The molecule with the highest activation z-score (3.7) day 1 *p.i*. was ABCB6 that is known to bind heme and porphyrins and plays a crucial role in heme synthesis^35,36^. Several other upstream regulators appeared to play a role in cell growth, DNA replication, and cancer (CDKN2A, TCF3, PARP1, let-7a-5p). Interestingly, medroxyprogesterone acetate and let-7 (miRNA) were activated on day 1 *p.i*, but were inhibited on day 4 *p.i*. (Supplementary Table S5). Similar to the observations in N67 infection, IL10R, SOCS1, TRIM24, and PTGER2 were also inhibited in mice day 1 infected with 17XNL. Another intriguing observation was the inhibition of GATA1 in both N67 and 17XNL infected mice (not in mice infected with N67 day 1 *p.i*.). Because GATA1 plays a critical role in erythropoiesis and in the switching of fetal hemoglobin to adult hemoglobin^37^, inhibition of GATA1 may suggest re-activation of fetal hemoglobin synthesis in the spleen.

To confirm the observations of altered gene expression and activation of upstream regulators after parasite infections, we extracted proteins from the spleens of infected mice day 1 and day 4 *p.i*. and detected protein expression and/or phosphorylation for some key molecules in interferon signaling pathways (Fig. 3b and Supplementary Fig. 6). The expression of MyD88, STAT1, and STAT2 increased after infection with both parasites day 1 and day 4 *p.i*. Phosphorylation of STAT2 increased in N67 infected mice only, whereas phosphorylation of STAT1 increased in mice infected with both parasites day 1 and day 4 *p.i*. Cytosolic RNA sensor MDA5 was greatly increased in N67 infected mice day 1 and day 4 *p.i*. (only slightly for 17XNL day 1). The expression of TRIF was increased day 4 *p.i*. with N67, and no obvious change in protein expression was observed for TLR4 and STING. These results confirm activation of interferon signaling, particularly IFN-I signaling mediated by MDA5 after N67 infection. Pathways mediated by MyD88, TRIF, and RIG-I are activated day 4 *p.i*. with both parasites.

### Universally inhibited Th1 and related pathways in mice infected with additional parasites

**Figure 4:**
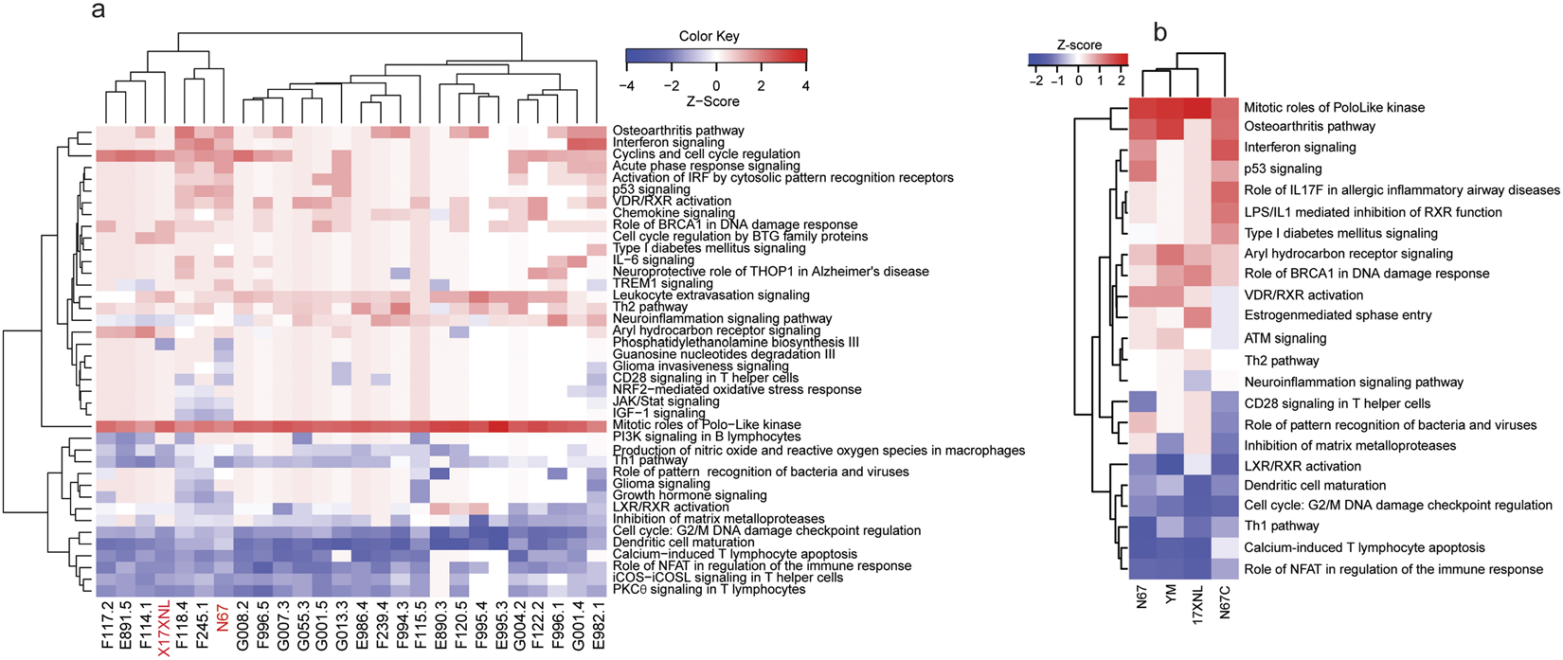
Hierarchical clusters of activated or inhibited pathways in mice infected with four *P. yoelii* strains and 24 genetic cross progeny of the 17XNL × N67 cross. (**a**) Clustering pathways from mice infected with 17XNL, N67, and their 24 progeny. The two parents are highlighted in red at the bottom. Pathway names are at the right of the figure. (**b**) Clustering pathways from mice infected with four *P. yoelii* strains/subspecies (bottom). 17XNL, *P. y. yoelii* 17XNL; YM, *P. y. yoelii* YM: N67, *P. y. nigeriensis* N67; N67C, *P. y. nigeriensis* N67C. Gene expression differences between infected and uninfected mice were compared using ANOVA using a 2.0-fold cutoff value. Ingenuity Pathway Analysis (IPA, Qiagen) was used to perform pathway enrichment (p ≤ 0.001) and activation/inhibition (z-score ≥ 2.0 for activation; z-score ≤− 2.0) analyses.

The observations of inhibited Th1 and related T cell pathways in mice infected with both N67 and 17XNL suggest that these pathways could represent commonly suppressed immune pathways after malaria parasite infection. To obtain additional data to support the observations, we extended our microarray analyses to mice infected with 24 progeny from the N67 × 17XNL cross^14^ and two additional parasite strains (N67C and YM, Fig. 1b). Again, mRNA samples were collected from the spleen of mice (3–5 each for each parasite) day 1 and day 4 *p.i*. (note the day 4 microarray data was reported for genetic linkage analysis previously^38^, but was not analyzed for gene expression) with the progeny and were hybridized to microarray chips as described above. At day 1 *p.i*., few significantly enriched pathways (2-fold cutoff in gene expression level) were detected among the progeny (data not shown). On day 4 *p.i*., the only activated (z-score ≥ 2.0) and significantly enriched (p ≤ 0.001) pathway in the parents and progeny (22 out of 24 progeny had Z-scores ≥ 2.0) was again the mitotic roles of Polo-like kinase (Fig. 4a and Supplementary Table S6). On the other hand, there were several pathways that were significantly (p < 0.001) enriched with negative z-scores for the majority of the parasites, including PKCθ signaling in T lymphocytes, iCOS-iCOSL signaling in T helper cells, Th1 pathway, calcium-induced T lymphocyte apoptosis, role of NFAT in regulation of the immune response, G2/M DNA damage checkpoint regulation, and DC maturation (Fig. 4a). The results from the progeny confirmed the suppression of Th1 and several T-cell related immune response pathways by day 4 *p.i*. as observed above.

To further confirm the activated and suppressed pathways in infection of more parasite strains, we performed another independent hybridization using mRNAs from mouse spleens infected with four *P. yoelii* strains (YM and N67C as well as N67 and 17XNL, Fig. 1b) day 4 *p.i*. First, the responses from mice infected N67C and YM that have unique disease phenotypes (Fig. 1b) provide additional data to support or reject the inhibited pathways. Second, the additional data from mice infected with 17XNL and N67 act as independent repeats. Using gene expression level of 2-fold cutoff, we also detected activation of mitotic roles of Polo-like kinase pathway in mice infected with YM and N67C (Fig. 4b). Again, pathways of LXR/RXR activation, DC maturation, G2/M DNA damage checkpoint regulation, Th1 activation, calcium-induced T lymphocyte apoptosis, and role of NFAT in regulation of the immune response were inhibited in mice infected with all of the four parasite strains.

### Clustering major differentially and commonly regulated pathways using PAGE method

**Figure 5:**
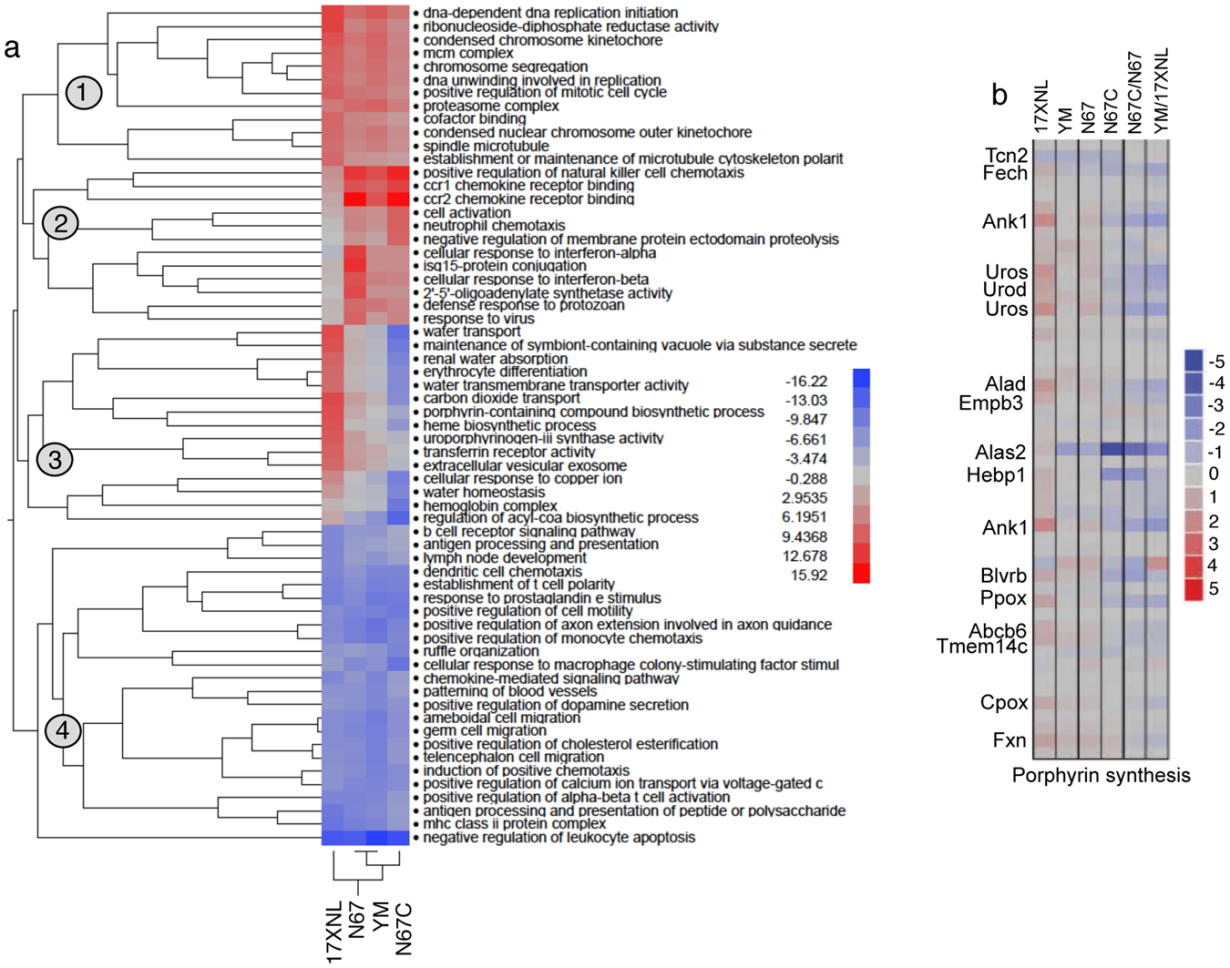
Hierarchical clusters of activated or inhibited pathways in mice infected with four *P. yoelii* strains. Gene expression significantly (adjusted p-value < −0.05) changed by ANOVA between infected and uninfected mice were used as input to Parametric Analysis of Gene Set Enrichment (PAGE). (**a**) Clusters of Gene Ontology Biological Process terms with corresponding z-scores from PAGE in color scale. (**b**) Log2 fold change of individual genes in the gene set “Porphyrin synthesis” (as indicated by individual strain infected vs. uninfected; or by the ratio of one strain to another). Gene IDs: *Fech*, ferrochelatase (or heme synthetase); *ank1*, ankyrin; *uros*, uroporphyrinogen III synthase; *urod*, uroporphyrinogen decarboxylase; *alad*, aminolevulinate dehydratase; *empb3*, erythrocyte membrane protein band 3; *alas2*, aminolevulinate-delta dehydratase 2; *hebp1*, heme binding protein 1; *blvrb*, biliverdin reductase b; *ppox*, protoporphyrinogen oxidase; *abcb6*, ATP-binding cassette subfamily B member 6; *Tmem14c*, transmembrane protein 14C; *cpox*, coproporphyrinogen oxidase; *fxn*.

We also analyzed the dataset from the four parasite strains day 4 *p.i*. using Parametric Analysis of Gene Set Enrichment (PAGE)^39^. ANOVA analysis and hierarchical clustering of the least squares means (Lsmean) of the treatment groups revealed 333 genes with probes that were significantly (adjusted p-value < 0.05) changed in expression in at least one of the parasite-infected groups compared with the uninfected controls (Supplementary Table S7). Subsequent clustering of the genes using PAGE showed four major groups of Gene Ontology (GO) functional pathways (Fig. 5a). First, pathways involved in DNA replication, chromosome segregation and movement, proteosome complex, and cell cycle regulation were upregulated in the parasite-infected mice, particularly those infected with 17XNL and YM, suggesting elevated DNA replication and cell division. Second, many pathways related to chemotaxis and interferon responses in mice infected with the three lethal parasites (YM, N67, and N67C) were upregulated. Among these, genes in pathways of positive regulation NK cell chemotaxis and CCR1/CCR2 chemokine receptor binding were highly expressed in mice infected with N67C and N67. Additionally, pathways involved in neutrophil chemotaxis, cell activation, and negative regulation of membrane protein ectodomain proteolysis were expressed at higher levels in the N67C infected mice, whereas pathways associated with IFN-I response such as 2′-5′-oligoadenylate synthetase activity, isg15-protein conjugation, cellular response to IFN-α/β, and defense response to protozoan were highly activated in N67-infected mice. Third, pathways involved in hematopoiesis, erythrocyte differentiation, heme/porphyrin synthesis, and water and CO2 transport were highly upregulated in mice infected with nonlethal 17XNL parasites, but downregulated in mice infected with N67C. The increased expression of these genes likely reflected active generation of new blood cells in mice infected with 17XNL. Finally, mice infected with all the four parasites had many downregulated pathways related to host immune response such as genes in antigen processing and presentation, DC and monocyte chemotaxis, positive regulation of αβT-cell activation, and negative regulation of leukocyte apoptosis, further supporting the observation of down-regulation of key immune pathways after *P. yoelii* infection.

Closer examination of the pathways related to erythrocyte differentiation and heme biosynthesis revealed many differentially regulated genes in these pathways. For example, genes in the porphyrin synthesis pathway, including ferrochelatase (*fech* or heme synthetase), ankyrin (*ank1*), uroporphyrinogen III synthase (*uros*), uroporphyrinogen decarboxylase (*urod*), aminolevulinate dehydratase (*alad*), aminolevulinate-delta dehydratase 2 (*alas2*), heme binding protein 1(*hebp1*), biliverdin reductase b (*blvrb*), protoporphyrinogen oxidase (*ppox*), ATP-binding cassette subfamily B member 6 (*abcb6*), transmembrane protein 14 C (*Tmem14c*), coproporphyrinogen oxidase (*cpox*), and frataxin (*fxn*), were up-regulated in 17XNL-infected mice, but down-regulated in N67C-infected mice (Fig. 5b). Further investigation on how the parasites stimulate (17XNL) or inhibit (N67C) host hematopoiesis may illuminate critical mechanism for understanding malaria-induced anemia.

### Dynamics of splenic cell populations after infection with four *P. yoelii* strains

**Figure 6:**
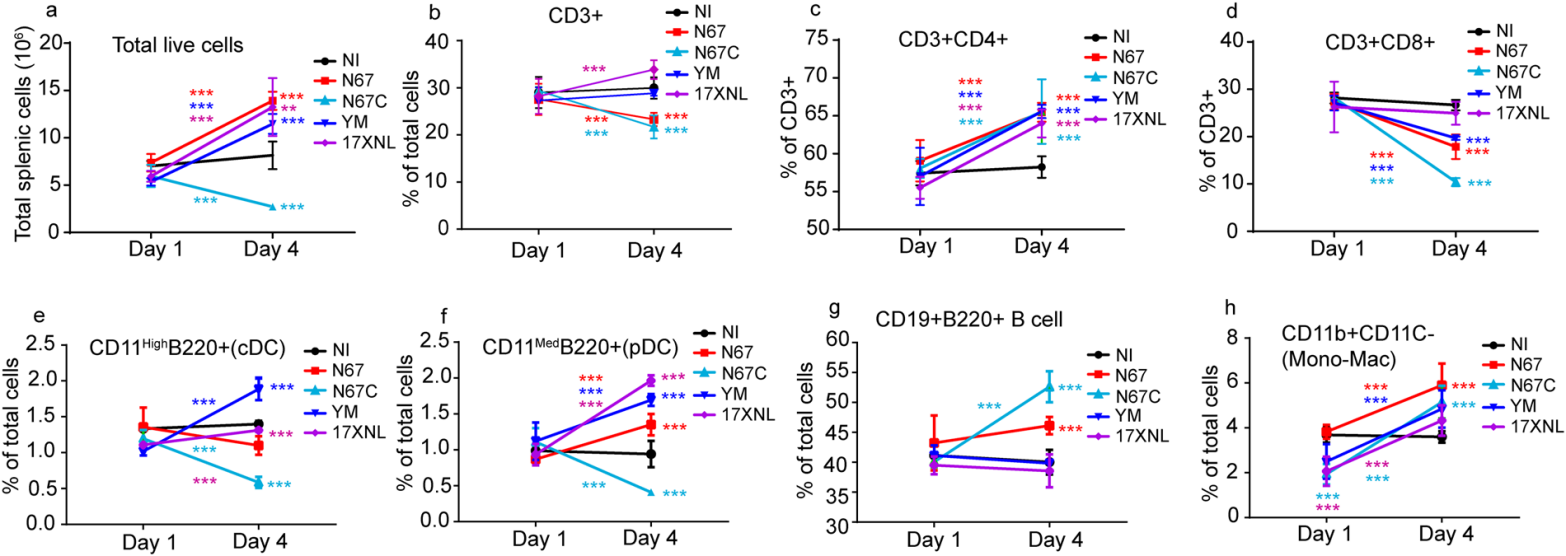
Splenic cell counts day 1 and day 4 after infections with four *P. yoelii* strains. (**a**–**h**) Different cell populations are as labeled. Cells from spleens infected with 17XNL, YM, N67, and N67C were stained with antibodies against various cell markers, as indicated, and were counted using flow cytometry. Cells from spleens of uninfected mice (NI) were also counted. Three mice in each group. Comparisons include cell counts on day 1 and day 4 or between strains on the same day. Significance was tested using Whitney-Mann U test; **p < 0.01; ***p < 0.001.

To investigate whether the reduced signals in the T cell related pathways were due to loss of cell populations in the spleen, we counted T-cells, B-cells, DC, and monocyte/macrophage from spleens of day 1 and day 4 infected mice. On day 1 *p.i*., all the cell counts were similar among the infected and uninfected groups (Fig. 6a–h). On day 4 *p.i*., mice infected with different parasites had different levels of cell populations, reflecting the differences in disease phenotypes. Mice infected with 17XNL, YM, and N67 displayed a similar trend of increased cell counts in total live splenic cells, CD4+ T cell, and plasmacytoid DC (pDC) (Fig. 6a,c,f). CD8+ T cells were the only cell population that was decreased in all infected mice day 4 *p.i*. (Fig. 6d). Day-4 B cell counts of the infected mice were either similar to that of uninfected (for 17XNL and YM infected) or increased (N67 and N67C), particularly those infected with N67C (Fig. 6g). Additionally, mice infected with N67C had decreased total live cell count, CD3+ T cell, CD8+ T cell, cDC, and pDC. Considering the increased day 4 CD4+ T and DC cells in infection with 2–4 of the 4 strains and similar day 1 cell counts for uninfected and all infected mice, the observations of inhibited pathways in mice infected with all the parasites such as Th1 pathway, calcium-induced T lymphocyte apoptosis, or role of NFAT in regulation of the immune response are unlikely due to loss of T cell or DC cell populations. Therefore, these pathways could represent common pathways that malaria parasites inhibit as one of their strategies to evade host immune clearance. Similarly, the activations of IFN-I pathways day 1 *p.i*. with N67 or 17XNL are due to increased gene expression because the day 1 cell populations are similar in all the infected groups and uninfected mice. Stimulation of these T-cell mediated pathways may re-activate host immune response to control malaria infection.

### Reversal of T cell pathway inhibition by anti-OX40 and anti-CD28 improves host survival

**Figure 7:**
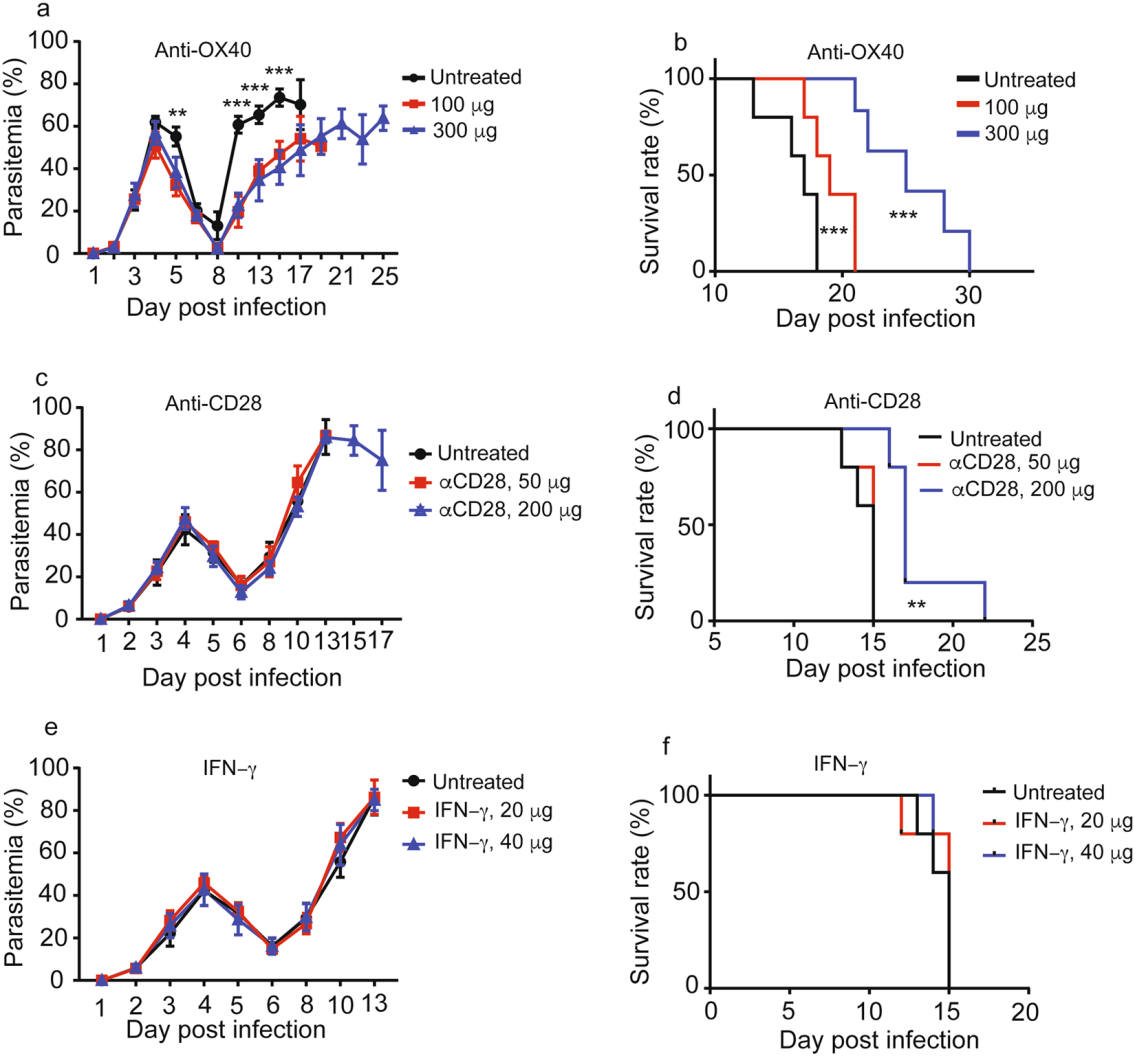
Modulation of inhibited T cell pathways improves host survival. (**a**) Parasitemia from N67-infected mice untreated or treated with anti-OX40 monoclonal antibody. (**b**) Host survival curves from N67-infected mice untreated or treated with anti-OX40 monoclonal antibody. (**c**,**d**) Parasitemia and host survival from N67-infected mice untreated or treated with anti-CD28 monoclonal antibody. (**e**,**f**) Parasitemia and host survival from N67-infected mice untreated or treated with IFN-γ. Significance was tested using Whitney-Mann U test; **p < 0.01; ***p < 0.001. The experiments were independently repeated once with almost the same results.

We next attempted to reverse the immune inhibition by injecting IFN-γ or antibodies that recognize some key receptors in T cell activation pathways. OX40 is a member of the TNF receptor family and is a costimulatory molecule that is expressed on activated CD4+ T and CD8+ T cells^40,41^. OX40 is known to activate PI3K/ PKB, NF-κB1, and NFAT pathways in antigen-dependent and antigen-independent signaling pathways, and anti-OX40 treatment promotes the proliferation and activation of lymphocytes through NFATc1^42^. We therefore injected anti-OX40 monoclonal antibody (100 μg and 300 μg per mouse, *i.v*., Biox Cell, West Lebanon, NH, Cat# BE0031) day 2 and day 3 *p.i*. to activate NFATc1 that was among the molecules inhibited after parasite infection. Injection of anti-OX40 antibody (5 mice per group) significantly reduced N67 parasitemia day 10 *p.i*. and increased host survival (Fig. 7a,b). Our microarray analysis showed down-regulation of CD86 that binds CD28 to activate T cells day 4 *p.i*.^43^. We next treated infected mice (5 mice per group) with anti-CD28 (50 μg and 200 μg) antibody day 2 and day 4, *i.v*. No change in N67 parasitemia was observed between anti-CD28 treated and untreated groups (Fig. 7c); however, better survival was observed in infected mice treated with 200 μg anti-CD28 antibody (Fig. 7d). For both anti-OX40 and anti-CD28, there appeared to be a dose effect, with better protection in the groups treated with higher antibody dosages. We also treated infected mice (5 mice per group) with IFN-γ that is known to stimulate T cell immunity; however, no obvious difference in parasitemia or host survival rate was observed between untreated and treated groups (Fig. 7e,f). Although these preliminary experiments did not eventually save the life of infected mice, the experiments support the results of our microarray analyses suggesting that reversal of malaria-inhibited T cell pathways can improve host survival and possibly better responses to vaccination.

## Discussion

Microarray-based genome-wide analyses of host responses to experimental infections of *P. falciparum*, *P. berghei*, and *P. yoelii* have been reported previously, and changes in gene expression pathways of inflammation, interferon responses, erythropoiesis, glycolysis, B cell activation, and immunoglobulin production were observed^12,22–24,44–46^. In addition, transcriptomics analysis has also been applied to investigate host immune responses in human challenge infections^47^. Pathways of PRR and IFN signaling, innate immune cell activation, inflammation, chemotaxis, antigen presentation, humoral response, apoptosis, glycolysis, and cell cycles were all detected after human malaria challenge infections. Additionally, genes in complement and toll-like receptor pathways and IFN-α/β signaling in NK cells were detected in natural human infections^48–50^. In another study of human infections comparing retinopathy-positive cerebral malaria (Ret + CM) and retinopathy-negative cerebral malaria (Ret − CM), increased transcripts of inflammatory cytokines were associated with Ret + CM, whereas Ret-CM was linked to higher levels of IFN-α2^51^. However, the majority of the studies of experimental infections used 1 or 2 parasite strains and focused on genes that were up-regulated after infections. Different parasite species and/or strains are known to cause different diseases and host responses^12^. Additionally, host responses to malaria infection are dynamic and time-dependent, e.g. different pathways or levels of cytokines are detected depending on the time of sampling. Studies using samples collected from patients generally do not know the time of infection, leading to variation in observations of host responses. Investigation and identification of pathways inhibited, as well as those activated, after malaria infections may provide useful information for effective activation of the inhibited host immune responses to improve parasite clearance, vaccine efficacy, and disease management. This current study investigates genome-wide host gene responses to infections with four *P. yoelii* subspecies/strains that cause very different disease phenotypes and 24 progeny of a genetic cross, with a major goal of identifying host response pathways universally inhibited by parasite infections. Shared inhibited pathways such as DC maturation, Th1 pathway, calcium-induced T lymphocyte apoptosis, and role of NFAT regulation of immune responses were detected in mice infected with all the parasites day 4 *p.i*. These pathways could represent pathways that are commonly suppressed in response to malaria infection.

Previous studies have suggested a requirement for Th1-like cellular responses for parasite clearance; however, inflammatory responses after Th1 activation need to be controlled properly in order to avoid cytokine storm and systemic disease^11^. The inhibition of the T cell pathways could explain the observation of human adult carriers showing limited parasite growth (relatively low parasitemia) without parasite clearance and disease symptoms. Inhibition of DC maturation and reduction of the subsequent T cell proliferative response was observed after binding of *P. falciparum* infected RBCs to CD36 and indirectly to CD51^52^. Similarly, infection of blood stage *P. yoelii* was shown to inhibit the previously established CD8+ T cell response induced by irradiated sporozoites^53^. The observed reduction in CD8+ T cells day 4 after infection with the four parasite strains is consistent with a negative effect on response of CD8+ T cells. Our observations of inhibition of Th1 and DC maturation pathways after infection with these *P. yoelii* parasite strains/subspecies and the 24 progeny suggest that these pathways are universally inhibited immune pathways early in malaria infection (day 4 *p.i*.). The inhibitions of these pathways are also supported by upstream regulator analysis: expression of genes encoding CD86, MHCII, CD1, IFNγR, IL27, PI3K, TIM3, and NFAT were down-regulated day 4 *p.i*. Clearly, the down-regulation of CD86 and MHCII on DC cells is critical for the inhibition of Th1 activation and related pathways because CD86 and MHCII of DC cells bind to CD28 and TCR, respectively, to activate T cells. Additionally, levels of STAT4 and IL-10 were increased day 4 *p.i*. with the parasites, which may also contribute to immune inhibition. Stimulation of expression of molecules in DC-T cell interactions may re-activate the suppressed Th1 pathways to better control parasitemia and disease. However, care should be taken to avoid over activation of inflammatory responses that could lead to severe disease.

The identification of inhibited pathways after malaria infection has significant implications in disease treatment and vaccine development. In a systematic analysis of protective immune responses to RTS,S vaccination and controlled human malaria challenge study, it was shown that protective immunity against *P. falciparum* could be achieved via high antibody titers or early innate and DC activation^54^. Adults in malaria endemic areas showed lower percentage in inducing sterile immunity or lower antibody titers than those from non-endemic areas, suggesting that the semi-immune individuals may have developed mechanisms adversely affecting (inhibiting) the development of a fully efficacious immune response^55,56^. Malaria infection has been shown to induce compromised DC function and T cell dysfunction^57,58^, and blockade of PD-L1 and the inhibitory receptor LAG-3 restored CD4+ T cell function in mice infected with *P. yoelii*^59^. PD-1 deficiency was shown to promote antigen specific T follicular helper (Tfh) cell expansion and improve host antibody response to immunization^60^. Further, higher PD-L2 expression on blood dendritic cells of *P. falciparum*-infected individuals was shown to be correlated with lower parasitemia^61^. In another study, Treg cells were found to impede protective immunity through cytotoxic-T-lymphocyte-associated protein-4 (CTLA-4), and targeting Treg cells or CTLA-4 could accelerate parasite clearance in mice^62^. Additionally, therapeutic OX40 ligation was shown to improve immunity and limit the growth of a nonlethal *P. yoelii* parasite^63^. Therefore, immune checkpoint blockade may become an effective strategy for preventing and treating infectious diseases, including malaria^64^. Our results showing reduced expression of CD40 and CD86, but unchanged expression of CD80 day 4 *p.i*., suggest potential induction of immune tolerance mediated by CD80-CTLA-4/PD-L1 interactions and activation of Treg^65,66^. Alternatively, DC maturation and impaired cross-presentation by CD8 DC after *P. berghei* ANKA infection have been reported^67^. Activation of immune pathways inhibited by malaria parasites could be an important approach for enhancing host immunity. However, whether the inhibition of these pathways leads to T cell dysfunction or exhaustion require further investigation.

Infections of malaria strains cause different disease phenotypes (parasitemia and mortality) and will also stimulate responses unique to each parasite strain^12^. Individuals with or without pre-priming with an adenovirus 35 (Ad35) vector before vaccination with RTS,S vaccine were found to have different host protection mechanisms^54^. Indeed, our data show that N67 infection induces a strong early IFN-I response, with activation of upstream regulators implicating recognition of parasite DNA/RNA (TLR3/7/8/9, cGAS, RIG-I, MDA5) and lipid/LPS (TLR2/4). The level (z-scores) of IFN-I response declined day 4 *p.i*., which is supported by down-regulated expression of many genes in the pathways. At a protein level, p-STAT2 (signal transducer and activator of transcription 2) was increased day 1 and then declined day 4 *p.i*. with N67, although MDA5 was still expressed at a high level. These results suggest increased IFN-Ι signaling day 1 *p.i*., but declined day 4 *p.i*. At the same time, indicators of inflammatory response such as production of IFN-γ, TNF-α, IL-1β, and IL-6 mediated by TLR/MyD88/NFκB were also activated after N67 infection, which may explain the pathology and host death later in infection. For 17XNL infection, weak signals of immune activation were observed day 1 and day 4 *p.i*. An upstream regulator of IFN-I with z-score = 2.16 was identified in mice day 1 *p.i*. with 17XNL, suggesting low level of IFN-I signaling. Many genes in interferon signaling pathways were also slightly up-regulated day 1 *p.i*. (Supplementary Table S3). Various ISG genes such as *Ifits*, *Mx1* and *Mx2* were detected in a previous microarray analysis after 17XNL infection^24^. The lower level of interferon responses compared to those of N67/N67C infection could be due to relatively slow growth and low parasitemia of the 17XNL infection, as suggested previously^24^. However, mice infected with YM had day 4 parasitemia (~50–60%) higher than those infected with N67 and N67C^13,16^, but with almost undetectable IFN response (Fig. 3d), suggesting that parasitemia is not the major factor determining host immune response. Indeed, infection with YM parasites was found to induce low levels of IFN-I only at 24 h *p.i*. through cytosolic sensors, which stimulated SOCS1 to inhibit MyD88 and prevent production of more IFN-I by TLR7 signaling^8^. In addition to a protective effect by a high IFN-I level induced during early infection^8,12^, IFN-I signaling has also been shown to impair Tfh differentiation or accumulation and antibody production^68,69^. The production of IFN-I could also contribute to the observed inhibition of T cell related pathways. The effects of IFN-I, either protective or damaging, on malaria infection are likely dependent on the timing, level, and duration of production.

Interestingly, pathways of heme and tetrapyrrole biosynthesis pathways were inhibited day 1 *p.i*. with 17XNL, but were activated day 4 *p.i*. These observations are consistent with a previous report of inhibition of erythroid transcription at low parasitemia (≤5%) and activation at high parasitemia (25%) after17XNL infection^24^. Activation of hematopoiesis will generate more reticulocytes that provide more cells for parasite growth because this parasite only invades reticulocytes^70,71^. The differential host responses to different parasite infections suggest that immuno-intervention or vaccination may need to consider variation in parasite genetics and the diverse disease symptoms they cause. Priming a host for strong T cell responses may lead to severe disease symptoms if the host is later exposed to parasites that can stimulate strong inflammatory responses.

Interrogation of mouse responses to infections with the 24 progeny provides additional support for the commonly inhibited pathways detected in mice infected with different strains/subspecies. The majority of inhibited pathways observed in the infections of parasite strains/subspecies were also suppressed in mice infected with the progeny. However, differences or even the opposite activation or inhibition of selected pathways were observed in mice infected with the progeny, consistent with generation of parasites with new genetic backgrounds.

In summary, our analyses detect unique and common host response pathways inhibited after *P. yoelii* infection as well as differentiated responses to individual parasites. As proof of principle, we also provide experimental data showing that modulation of inhibited T cell activation pathways can improve host survival. These pathways can be explored to enhance parasite clearance and vaccine development. On the other hand, variation in parasite genetic background and strain specific responses will need to be considered in disease treatment and management. This study provides important information for designing strategies to improve anti-malaria immunity and vaccine efficacy.

## Methods

### Ethics statement

All animal procedures for this study were performed in accordance with the protocol approved (approval #LMVR11E) by the Institutional Animal Care and Use Committee (IACUC) at the National Institute of Allergy and Infectious Diseases (NIAID) following the guidelines of the Public Health Service Policy on Humane Care and Use of Laboratory Animals and AAALAC. All mice were maintained under pathogen-free conditions.

### Animals and malaria parasites

Inbred female C57BL/6 mice, aged 6–8 weeks, were purchased from Charles River Laboratory. *P. y. yoelii* 17XNL, *P. y. yoelii* YM, *P. y. nigeriensis* N67, *P. y. nigeriensis* N67C, and 24 progeny from the N67 × 17XNL cross were described previously^13,14^. An inoculum containing 1 × 10^6^ iRBC suspended in 100 ml of phosphate-buffered saline, pH 7.4, from donor mice was injected intravenously into each experimental C57BL/6 mouse to initiate infection. Three to six mice as a group were infected with each parasite, and parasitemia were monitored by microscopic examination of Giemsa-stained thin tail blood smears. All the parasites were tested and were free of mycoplasma infection.

### RNA samples for microarray hybridization

Spleens from day 1 and day 4 infected or uninfected mice were removed and immediately preserved in RNA Later solution (Qiagen, Hilden, Germany). RNA was isolated using a RNeasy mini kit following the manufacturer’s protocol (Qiagen). RNA quality was determined using Agilent Bioanalyzer as well as NanoDrop UV spectroscopy.

### Microarray hybridization

RNA samples were individually amplified and labeled from 500-ng input using the Illumina TotalPrep RNA Amplification kit (Applied Biosystems, Foster City, CA). Biotinylated cDNA was hybridized to Illumina MouseRef-8 v2.0 Expression BeadChip Illumina MouseWG-6 v2.0 expression beadchip (GEO accession no. GPL6885 and GPL6887) having 25,697 (or 45,281) unique probes using reagents provided. Amplification and labeling of RNA samples were performed using the Illumina TotalPrep RNA Amplification kit (Applied Biosystems). Illumina HiScan was used for chip imaging and Genome Studio software for data extraction. The data discussed in this publication have been deposited in NCBI’s Gene Expression Omnibus and are accessible through GEO Series accession number GSE114718 (https://www.ncbi.nlm.nih.gov/geo/query/acc.cgi?acc=GSE114718) and GSE63611 (https://www.ncbi.nlm.nih.gov/geo/query/acc.cgi?acc=GSE63611).

### Microarray data analysis

Quantile normalization, mixed-effect ANOVA, *P*-value adjustments, and gene set enrichment analysis^39^ were performed using JMP/Genomics software (SAS Institute). Gene sets were derived from Gene Ontology term assignments available from National Center for Biotechnology Information. Pathway and upstream regulators were analyzed using Ingenuity Pathway Analysis (IPA, QiaGen). *P*-value was calculated using a Right-Tail Fisher’s Exact Test, reflecting the association or overlap between a set of significant molecules from the experiment and a given pathway. Z-score is a measure to infer the activation states of predicted transcriptional regulators. The definition of z-score can be found at (http://pages.ingenuity.com/rs/ingenuity/images/0812%20upstream_regulator_analysis_whitepaper.pdf). In practice, z‐scores greater than 2 or smaller than ‐2 can be considered significant.

### Western Blot

Western blot was performed as described previously^12^. Briefly, infected or uninfected mouse spleens were removed day 1 and day 4 *p.i*. and immediately frozen in liquid nitrogen. Total proteins were separated by 4–20% SDS-polyacrylamide gel electrophoresis (SDS/PAGE), transferred to a PVDF membrane (Roche Diagnostics), and probed with indicated primary antibodies and corresponding secondary antibody before chemiluminescent detection (SuperSignal West Pico Chemiluminescent Substrate; Pierce). Antibodies were purchased from Cell Signaling (Danvers, MA; anti-MyD88, Cat # 4283; anti-RIG-I Cat# 3743; anti-TLR4, Cat# 14358, anti-STING, Cat# 13647; anti-MDA5, Cat#5321), Abcam (Cambridge, United Kingdom; anti-p-STAT2, Cat# ab53132; anti-STAT2, Cat# ab231184; anti-p-STAT1, Cat# ab29045; anti-STAT1, Cat# ab3987; anti-TRIF, Cat# ab180689) and Sigma-Aldrich (St. Louis, MO; anti-β-actin, Cat# A2228).

### Splenocyte Staining

Mouse splenocytes were stained with Zombie NIR in PBS (Biolegend, Cat# 423106) at room temperature for 20 minutes. The cells were incubated with anti-mouse CD16/CD32 (clone 93; eBiosciences) in PBS containing 2% dry milk to block FcγRII/III at 4 °C for 30 min. Surface staining was performed with anti-CD3e (clone 145-2C11), anti-CD4 (clone rm4–5), anti-CD8α (clone 53-6.7), anti-CD19 (clone 6D5), anti-B220 (clone RA3–6B2), anti-CD11c (clone N418), anti-CD11b (clone M1/70), and anti-ly6G (clone 1A8) purchased from Biolegend (San Diego, CA).

### Treatment of mice

To activate pathways that were inhibited by malaria parasites, anti-CD28 (Biox Cell, Cat# BE0015-1), anti-OX40 (Biox Cell, Cat# BE0031), IFN-γ (Peprotech Inc, Cat# 315-05), their corresponding isotype antibodies, and PBS were administered into mice *i.v*. on day 2, day3, or day 4 post infected with 1 × 10^6^ N67 parasites. Parasitemia were monitored by microscopic examination of Giemsa-stained thin tail blood smears.

## Electronic supplementary material

Supplementary figures and tables

Dataset 1

Dataset 2

Dataset 3

Dataset 4

Dataset 5

Dataset 6

Dataset 7
