# Supplementary material for: Detection of host pathways universally inhibited after Plasmodium yoelii infection for immune intervention
Source: Sci Rep. 2018 Oct 16;8:15280. doi: 10.1038/s41598-018-33599-1 (PMC6191451; doi:10.1038/s41598-018-33599-1)
Supplement: Supplementary file 1 — Supplementary figures and tables [file 41598_2018_33599_MOESM1_ESM.pdf]

Supplementary Information

Detection of host pathways universally inhibited after *Plasmodium yoelii* infection for immune intervention

Lu Xia[1,2*], Jian Wu[1*], Sittiporn Pattaradilokrat[1,3], Keyla Tumas[1], Xiao He[1], Yu-chih Peng[1], Ruili Huang[4], Timothy G. Myers[5], Carole A. Long[1], Rongfu Wang[6], Xin-zhuan Su[1#]

[1]Malaria Functional Genomics Section, Laboratory of Malaria and Vector Research, National Institute of Allergy and Infectious Disease, National Institutes of Health, Bethesda, MD 20892-8132, USA;

[2]State Key Laboratory of Medical Genetics, Xiangya School of Medicine, Central South University, Changsha, Hunan 410078, The People's Republic of China;

[3]Department of Biology, Faculty of Science, Chulalongkorn University, Bangkok 10330, Thailand;

[4]National Center for Advancing Translational Sciences, National Institutes of Health, Bethesda, MD 20892-8132, USA;

[5]Genomic Technologies Section, Research Technologies Branch, National Institute of Allergy and Infectious Diseases, National Institutes of Health, Bethesda, MD 20892-8132, USA;

[6]Center for Inflammation and Epigenetics, Houston Methodist Research Institute, Houston, TX 77030, USA

Supplementary tables.

Supplementary Table S1. Top 10 most significantly changed pathways day 1 (24h) and day 4 post infection with *Plasmodium y. nigeriensis* N67 and *P. y. yoelii* 17XNL.

Supplementary Table S2. Selected enriched and activated or inhibited pathways day 1 and day 4 after infection with *Plasmodium y. nigeriensis* N67 or *P. y. yoelii* 17XNL, sorted by Z-score.

Supplementary Table S3. Gene expression levels in selected pathways differentially regulated between day 1 and day 4 post infections with *Plasmodium y. nigeriensis* N67 *or P. y. yoelii* 17XNL.

Supplementary Table S4. Gene expression levels in heme biosynthesis pathways differentially regulated between day 1 and day 4 post infection with *Plasmodium y. yoelii* N67.

Supplementary Table S5. Upstream regulators of activated or inhibited signaling pathways day 1 and day 4 post infection with *Plasmodium y. nigeriensis* N67 or *Plasmodium y. yoelii* 17XNL.

Supplementary Table S6. Z-scores of enriched pathways after infection with *Plasmodium y. nigeriensis* N67, *Plasmodium y. yoelii* 17XNL, and their 24 progeny.

Supplementary Table S7. Genes that are significantly differentially expressed in mice infection four *P. yoelii* strains.

Supplementary Figures

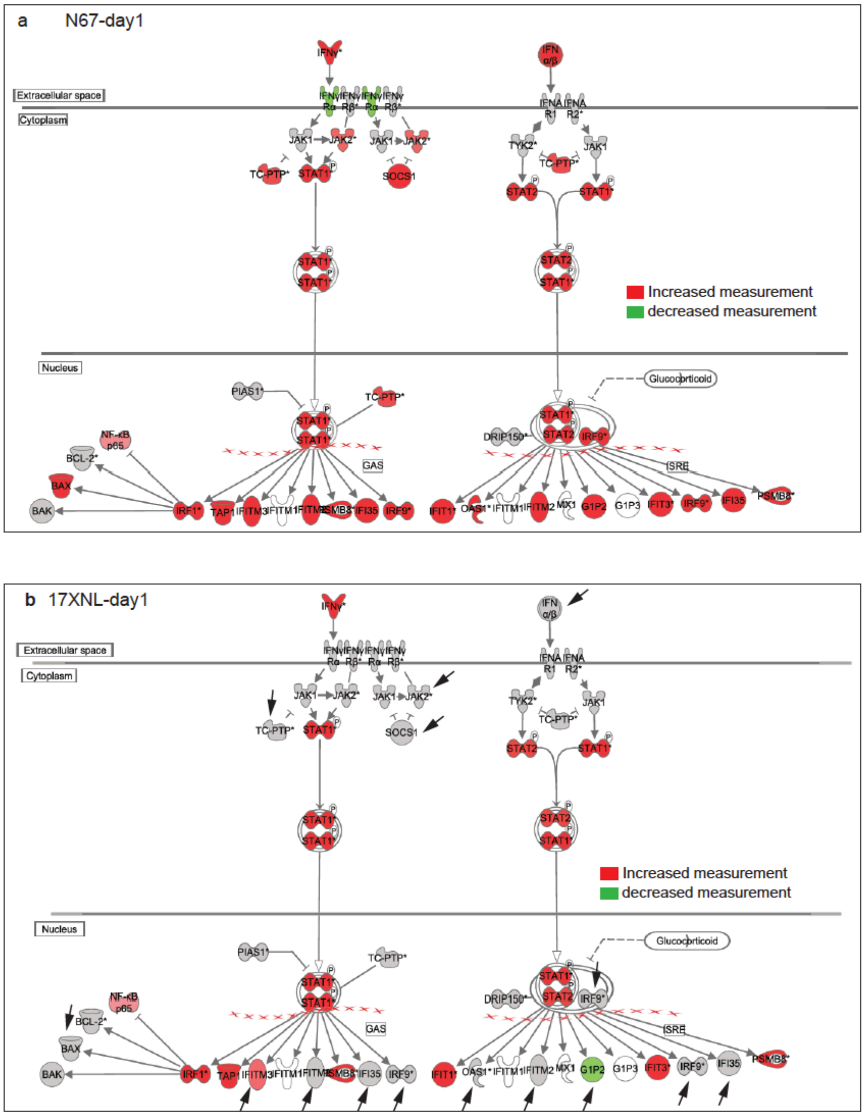

Supplementary Figure S1. Comparison of interferon signaling (IFN-α/β and IFN-γ) pathways in mice day 1 post infection with N67 (**a**) or 17XNL (**b**). Genes differentially expressed in infected and uninfected mice with a cutoff value of 1.2-fold or larger changes were selected for analysis of gene interaction networks that were generated through the use of IPA (QIAGEN Inc., https://www.qiagenbio- informatics.com/products/ingenuity-pathway-analysis). (**a**), Differentially regulated genes in interferon signaling pathways in mice day 1 post infection with

N67 parasite. (**b**), Differentially regulated genes in interferon signaling pathways in mice day 1 post infection with 17XNL parasite. The names of molecules are as indicated. Red, up-regulated genes; green down-regulated genes. Darker colors suggest higher up- or down-regulated, respectively. Arrows point to genes with lower expression in 17XNL infected mice.

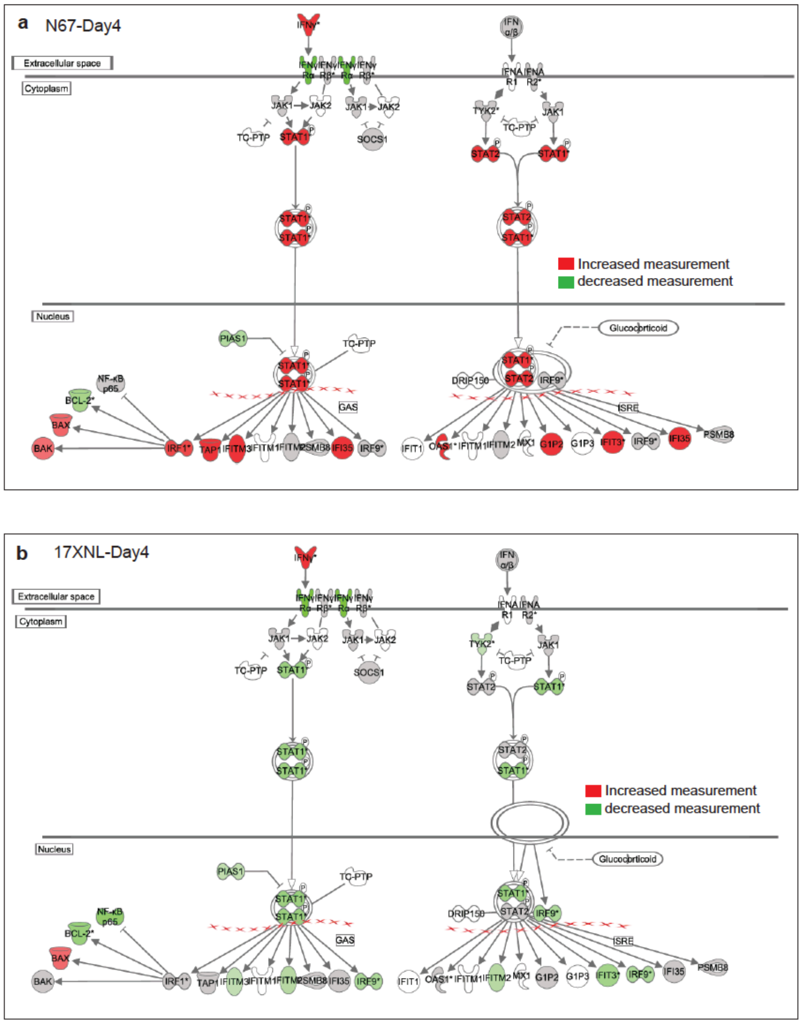

Supplementary Figure S2. Comparison of interferon signaling (IFN-α/β and IFN-γ) pathways in mice day 4 post infected with N67 (**a**) or 17XNL (**b**). Genes differentially expressed in infected and uninfected mice with a cutoff value of 1.2-fold or larger changes were selected for

analysis of gene interaction networks that were generated through the use of IPA (QIAGEN Inc., https://www.qiagenbio- informatics.com/products/ingenuity-pathway-analysis). (**a**), Differentially regulated genes in interferon signaling pathways in mice day 4 post infection with N67 parasite. (**b**), Differentially regulated genes in interferon signaling pathways in mice day 4 post infection with 17XNL parasite. The names of molecules are as indicated. Red, up-regulated genes; green down-regulated genes. Darker colors suggest higher up- or down-regulated, respectively.

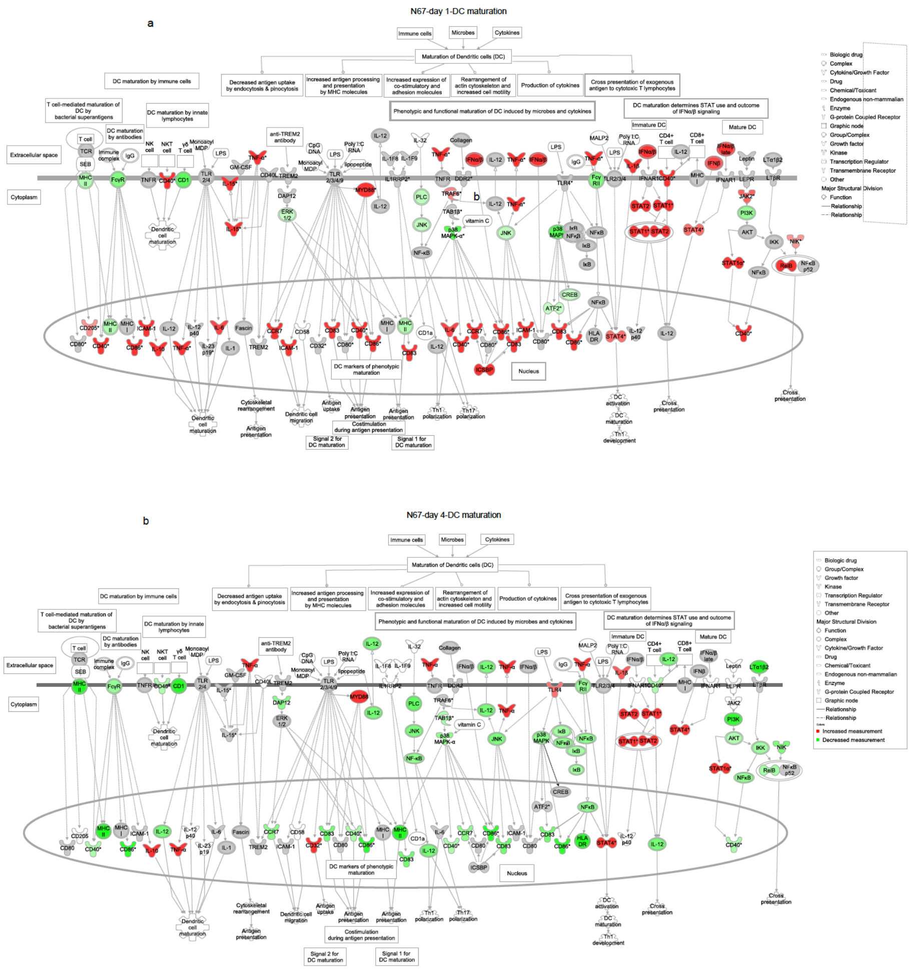

Supplementary Figure S3. Expression regulation of genes in dendritic cell (DC) maturation pathway day 1 and day 4 post infection with N67. Genes differentially expressed in infected and uninfected mice with a cutoff value of 1.2-fold or larger changes were selected for analysis of gene interaction networks that were generated through the use of IPA (QIAGEN Inc., https://www.qiagenbio- informatics.com/products/ingenuity-pathway-analysis). (**a**), Expression and interaction network of key genes in DC maturation pathway day 1 post infection with N67. (**b**), Expression and interaction network of key genes in DC maturation pathway day 4 post infection with N67. Red, up-regulated genes; green, down-regulated genes. Darker colors

suggest higher up- or further down-regulated genes, respectively. Symbols for molecular functions are as presented in the side box.

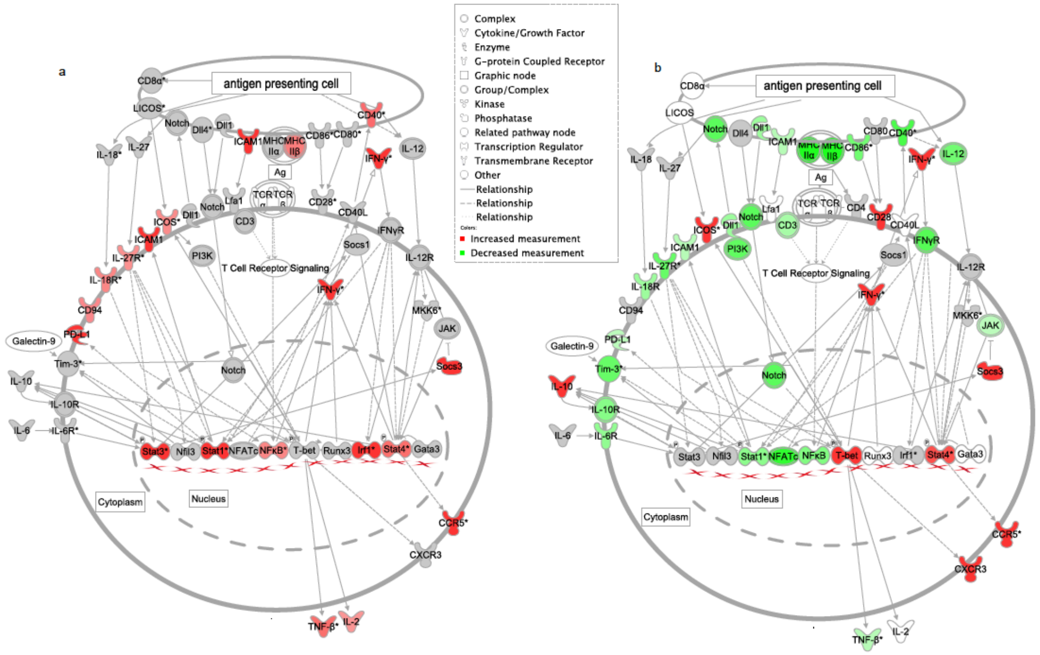

Supplementary Figure S4. Expression regulation of genes in Th1 pathway day 1 and day 4 post infection with 17XNL. Genes differentially expressed in infected and uninfected mice with a cutoff value of 1.2-fold or larger changes were selected for analysis of gene interaction networks that were generated through the use of IPA (QIAGEN Inc., https://www.qiagenbio-informatics.com/products/ingenuity-pathway-analysis). **(a)**, Expression and interaction network of key genes in Th1 pathways day 1 post infection with 17XNL. **(b)**, Expression and interaction network of key genes in Th1 pathways day 4 post infection with 17XNL. Red, up-regulated genes; green, down-regulated gene. Darker colors suggest higher up- or further down-regulated genes, respectively. Symbols for molecular functions are as presented in the side box.

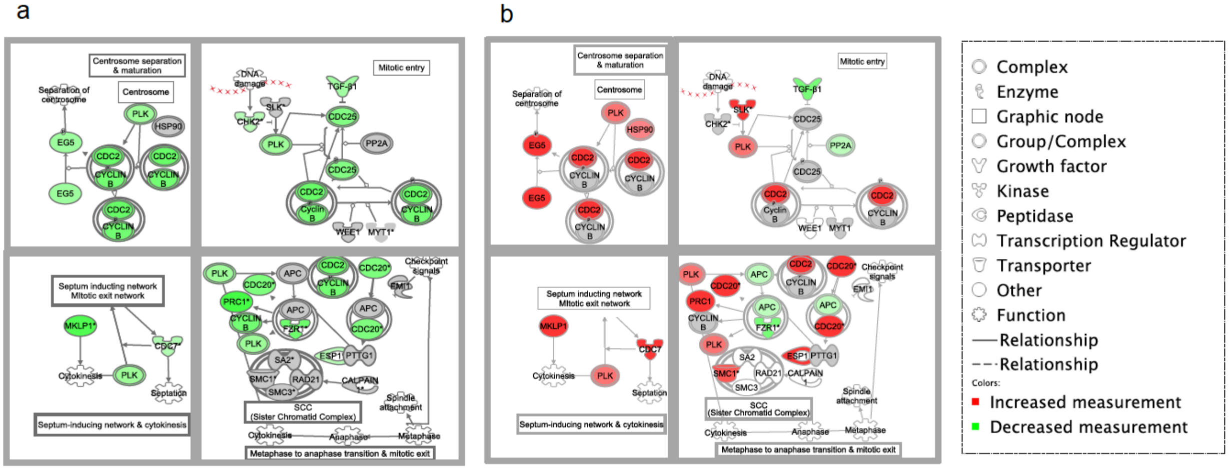

Supplementary Figure S5. Expression regulation of genes in mitotic roles of Polo-like kinase pathway day 1 and day 4 post infection with N67. Genes differentially expressed in infected and uninfected mice with a cutoff value of 1.2-fold or larger changes were selected for analysis of gene interaction networks that were generated through the use of IPA (QIAGEN Inc., https://www.qiagenbio- informatics.com/products/ingenuity-pathway-analysis). (**a**), Expression and interaction network of key genes in mitotic roles of Polo-like kinase pathway day 1 post infection with N67. (**b**), Expression and interaction network of key genes in mitotic roles of Polo-like kinase pathway day 4 post infection with N67. Red, up-regulated genes; green, down-regulated gene. Darker colors suggest higher up- or further down-regulated genes, respectively. Symbols for molecular functions are as presented in the side box.

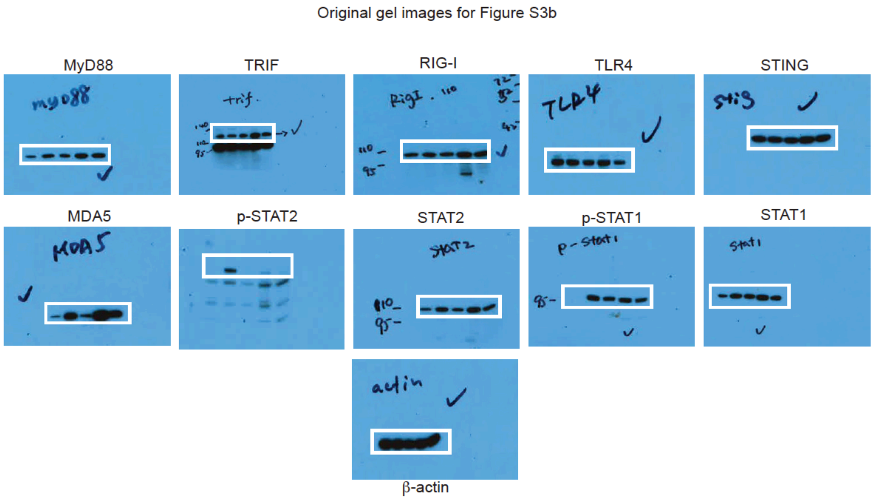

Supplementary Figure S6. Original Western blot images for panels in Figure 3b. The areas within the white boxes are cropped and presented.
